# Experiences of early graduate medical students working in New York hospitals during the COVID-19 pandemic: a mixed methods study

**DOI:** 10.1186/s12909-021-02543-9

**Published:** 2021-02-18

**Authors:** Harrison D. Pravder, Liana Langdon-Embry, Rafael J. Hernandez, Nicholas Berbari, Steven P. Shelov, Wendy L. Kinzler

**Affiliations:** 1 Renaissance School of Medicine, Stony Brook University, HSC T4-147, Stony Brook, NY 11794 USA; 2 NYU Long Island School of Medicine, NYU Langone Hospital – Long Island, 222 Station Plaza, Fifth Floor, Suite 510, Mineola, NY 11501 USA

**Keywords:** COVID-19, Early graduation, Medical students, Undergraduate medical education, New York

## Abstract

**Background:**

The coronavirus disease 2019 (COVID-19) pandemic presented the world with a sudden need for additional medical professionals. Senior medical students were identified as potential workers and many worldwide graduated early to serve as Junior Physicians in hospitals. The authors sought to identify factors that informed the decision to work, describe experiences in this capacity, and elucidate benefits for trainees.

**Methods:**

The investigators conducted a mixed-methods observational cohort study of early medical graduates eligible to work as Junior Physicians at two New York medical centers in April/May 2020 during an initial surge in COVID-19 hospitalizations. Graduates were surveyed, and a sample of Junior Physicians participated in a focus group. Survey responses of those who worked were compared to those who did not. Focus group responses were transcribed, coded, and thematically analyzed.

**Results:**

Fifty-nine graduates completed the study methods and 39 worked as Junior Physicians. Primary reasons for working included duty to help (39 [100%]), financial incentive (32 [82%]), desire to learn about pandemic response (25 [64%]), and educational incentive (24 [62%]). All had direct contact with COVID-19 patients, believed working was beneficial to their medical training, and were glad they worked. None contracted a symptomatic infection while working. Compared with non-Junior Physicians, Junior Physicians reported increased comfort levels in completing medical intern-level actions like transitions of care functions, such as writing transfer notes (*P* < 0.01), writing discharge orders (*P* = 0.01), and providing verbal sign out (*P* = 0.05), and they reported more comfort in managing COVID-19 patients. Sixteen themes emerged from the focus group and were placed into four categories: development of skills, patient care, safety, and wellness.

**Conclusions:**

Senior medical students chose to work as Junior Physicians for both personal and educational reasons. Experiences were beneficial to trainees and can inform future innovations in medical education.

**Supplementary Information:**

The online version contains supplementary material available at 10.1186/s12909-021-02543-9.

## Background

The New York metropolitan area was one of the first and most affected by the coronavirus disease 2019 (COVID-19) pandemic in the United States, calling for an immediate need for additional physicians and medical professionals to serve the community. This resulted in deployment of providers to New York from out of state, reassignment of medical personnel from other specialties, and innovative utilization of medical trainees in several capacities [1]. Medical students became pivotal participants in this response, as students took roles such as assembling personal protective equipment (PPE), staffing COVID-19 public information lines, and providing childcare for health care workers [2–4]. Thirteen medical schools in New York also provided final year medical students the opportunity to graduate early and serve in a physician role in order to address expected or ongoing staff shortages [1]. Globally, medical students graduated early in Canada, Denmark, Germany, Ireland, Italy, Iran, and the United Kingdom to serve in the pandemic as well [5–10].

Calling for medical student help during a crisis is not unique to this moment in time. History has sought medical student assistance during the Second World War when an accelerated three-year pathway was offered in the United States in order to compensate for staff sent overseas [11, 12] and in the 1918 Spanish Influenza when medical students cared for patients in a role akin to physicians [13]. During the 1952 poliomyelitis epidemic in Copenhagen, hundreds of medical students provided round-the-clock manual ventilation to intubated patients [14]. Unfortunately, few firsthand accounts of these activities are available.

Our institutions represent two New York academic centers where early medical graduates worked as paid practitioners under a new classification of medical professional in the hospital (henceforth known as “Junior Physicians” or “JPs”). Early graduates were voluntarily appointed in concert with guidelines issued by the Coalition for Physician Responsibility, ensuring the ability to opt out of such an experience and with provisions guaranteeing proper time to begin graduate medical education (GME) appointments without delay [15].

The objective of this study was to identify factors that informed the decision of early medical graduates to work as JPs and describe their experiences while working during the COVID-19 pandemic. The description of these elements will provide a framework for course of action should a similar situation present itself in the near or distant future and serve as a valued historical reminder. It adds to the literature on medical education innovations in the pandemic by describing and evaluating medical student early graduation and deployment to inpatient COVID-19 care [16, 17]. In addition, much can be learned about how the roles and responsibilities of these JPs impacted their transition to residency, potentially driving future educational changes.

## Methods

We conducted a mixed-methods observational cohort study employing multiple surveys and a focus group of early medical student graduates eligible to work at NYU Langone Hospital – Long Island (NYUW, Mineola, NY, USA; formerly known as NYU Winthrop Hospital) and Stony Brook University Hospital (SBUH, Stony Brook, NY, USA) during the early stages of the COVID-19 pandemic in New York. We sought to identify factors relevant in the decision to work as a JP, describe the experiences of JPs, and elucidate lessons and amendable circumstances to improve future experiences. First, quantitative surveys were distributed to assess early graduate experiences and compare objective measures between JPs and Non-JPs. Then, a focus group was held to further expand on identified topics and establish a deeper understanding of the JP experience. The study protocol was approved by the NYU Langone Health Institutional Review Board prior to recruitment (S20–00715).

### Subjects

On April 8, 2020, all medical students at Renaissance School of Medicine (Stony Brook, NY, USA) graduated early and were given the option to work as JPs. Those who chose to work were provided an orientation which included COVID-19 education, personal protective equipment simulations, telehealth training, and electronic health record training. JPs were then incorporated into the hospital workforce under the supervision of the Department of Medicine faculty. Roles included assisting the medical and intensive care unit teams, phlebotomy, proning, and assisting the line placement team. JPs were placed into the hospital reflecting the clinical campus where they had been students for the predominance of their clinical education, either NYUW or SBUH. Early graduates from other local medical schools who had matched to NYUW for their graduate medical education were offered a JP position at NYUW under certain circumstances and eligible for inclusion if they worked as JPs (*n* = 3).

All eligible early graduates (*n* = 122) were offered informed consent via email. They were divided into those graduates who chose to work as JPs and those who did not (Non-JPs).

### Evaluative surveys

Five unique surveys were distributed electronically through the Qualtrics platform (Provo, Utah) in May 2020. Given the sensitive nature of discussion, participation was voluntary, anonymous, and participants could choose to skip select survey questions. Prior to enrollment, graduates were informed that the decision to enroll would not affect their residency training, employment, or academic status. Survey tools were piloted among study investigators prior to dissemination.

Initially, we asked all early graduates to fill out a survey aggregate, composed of a demographics questionnaire, initial experience survey, and three other surveys (Intern Skills, COVID Care, and Burnout Assessment: disseminated May 15, 2020). The Intern Skills survey was an 18-item tool that gauged self-reported confidence or comfort in important skills to hone for the medical internship year, as deemed so by a majority of Internal Medicine program directors [18]. The COVID Care survey contained 13 questions that evaluated the comfort-level of early graduates in managing and monitoring patients with COVID-19 infection. All questions in these surveys were on a 5-point Likert scale. The Burnout Assessment scale was a single-item measure previously developed and validated to evaluate burnout levels [19].

Those who worked as JPs and filled out the initial surveys were invited on May 29, 2020 to complete the Recap survey, which sought to further understand JP experiences. The survey contained up to 27 questions, including open-ended, yes/no, and 5-item Likert scale items. All evaluative surveys are provided in Supplementary file 1.

### Focus group

JPs who completed all surveys were eligible to participate in a focus group to discuss their experiences. The one-hour virtual session was conducted using the Zoom application (San Jose, CA) on June 2, 2020 and facilitated by a faculty member (NB). It included 9 questions, which were informed based on survey responses. Audio was recorded and transcribed. Confidentiality was maintained by using generic names for each participant, disabling the video feature, and maintaining the transcript in a secure online location. The focus group script is provided in Supplementary file 2.

### Data analysis

Descriptive statistics were obtained for relevant study variables. Categorical data were compared using Fisher’s exact test. Continuous data were compared using t-tests for independent samples. Likert data were compared using Wilcoxon rank sum test. Cramer’s V is reported as an assessment of effect size [20]. This calculation is used to define strength of association after statistical significance is determined. Cramer’s V of 0 notes no relationship, values 0.10 to 0.29 show a small association, values 0.30 to 0.49 show a moderate association, and values greater than 0.50 show a large association. Focus group and textual responses were analyzed qualitatively by three authors (HDP, LLE, and RJH). Coding of phrases and texts was performed using the general inductive approach to qualitative evaluation for identification of key themes [21]. Final themes were agreed on by HDP, LLE, and RJH.

## Results

### Participants

One hundred twenty-two early graduates were eligible for inclusion. Sixty-seven (55%) provided consent. Eight of these students insufficiently completed the initial survey and their answers were omitted from analysis, leaving 59 participants in the analyzed data pool; of these, 39 worked as JPs. Table 1 displays the demographic data for these individuals.

**Table 1:** Baseline characteristics of early graduate medical students who worked as COVID-19 junior physicians and those who did not, May 2020^a^

Characteristic:	Junior physician (*n* = 39)	Non-junior physician (*n* = 20)	*P*^b^	*Cramer’s V*^*c*^
Age, mean [SD], years	28 [2]	27 [2]	0.52	–
Gender, n (%)			0.71	–
Female	21 (54)	10 (50)		
Male	16 (41)	10 (50)		
Other	2 (5)	0		
Matched GME specialty, n (%)			*0.02*	*0.38*
Surgical	19 (49)	7 (35)		
Medical	14 (36)	3 (15)		
Hospital-based	6 (15)	10 (50)		
Distance to matched residency program, n (%)			0.58	–
< 50 miles	20 (51)	7 (35)		
50 to 100 miles	5 (13)	2 (10)		
101 to 200 miles	3 (8)	2 (10)		
> 200 miles	11 (28)	9 (45)		
Home cohabitants. n (%)			*0.02*	*0.42*
None	22 (56)	6 (32)		
Significant other (SO)	11 (28)	3 (15)		
Parents	6 (15)	8 (42)		
Children and SO or parents	0	2 (11)		
COVID-19 status prior to work period, n (%)				
Tested positive	5 (13)	0	*0.16*	–
Family encouragement to work as JP, n (%)			*0.02*	*0.35*
Encouraged	7 (18)	0		
Discouraged	10 (26)	11 (55)		
Neither encouraged nor discouraged	22 (56)	9 (45)		
Felt Pressure to Work as JP, n (%), yes	6 (15)	9 (45)	*0.03*	*0.32*

*Abbreviations: COVID-19* Coronavirus disease 2019, *GME* Graduate Medical Education, *IQR* interquartile range, *JP* Junior Physician, *SD* standard deviation, *SO* significant other

^a^Includes sample of early medical school graduates eligible to work as Junior Physicians at NYU Langone Hospital – Long Island and Stony Brook University Hospital during the initial peak in cases in the COVID-19 pandemic in New York State

^b^Categorical data were compared using the Fisher exact probability test. Continuous data (age) was compared using t tests for independent samples with an assumption of unequal variance

^c^Cramer’s V standard interpretation for effect size: small association [0.10–0.29]; medium association [0.30–0.49]; large association [0.50–1.00]

At the time of recruitment, there were no differences regarding age, gender, and distance to matched GME program between the two groups. JPs had a higher proportion of graduates who matched to GME programs in surgical specialties (49%, *n* = 19 versus 35%, *n* = 7: general surgery, obstetrics and gynecology, surgical subspecialty) and medical specialties (36%, *n* = 14 versus 15%, *n* = 3: family medicine, internal medicine, medicine/pediatrics, pediatrics) but not hospital-based specialties (15%, *n* = 6 versus 50%, *n* = 10: anesthesiology, emergency medicine, neurology, psychiatry, radiology). JPs had taken an average of 21 weeks (standard deviation *n* = 6 weeks) of clinical coursework between the end of their core clerkships and graduation. Fifteen percent of JPs (*n* = 6) volunteered outside of the hospital compared with 25 % of Non-JPs (*n* = 5) (see Supplementary file 3 for methods in which graduates volunteered).

### Evaluative surveys

Participants chose various reasons for why they decided to work (or not work) as a JP. Common motivators to work included duty to help (*n* = 39, 100%), financial incentive (*n* = 32, 82%), desire to learn about pandemic response (*n* = 25, 64%), educational incentive (*n* = 24, 62%), opportunity to work with peers (*n* = 24, 62%), and head start on preparation for residency (*n* = 12, 31%). Non-JPs (*n* = 20) described various concerns for why they chose not to participate, primarily health worries (for family: *n* = 12, 60%; for self: *n* = 6, 30%; possible lack of PPE: *n* = 10, 50%), time constraint due to residency start date (*n* = 7, 35%), and concern about burnout (*n* = 4, 20%). A majority of JPs were worried about availability of PPE prior to working in the hospital (*n* = 21, 54%). JPs believed that their orientation was sufficient in preparing them for their role (*n* = 30, 77%).

**Fig. 1:**
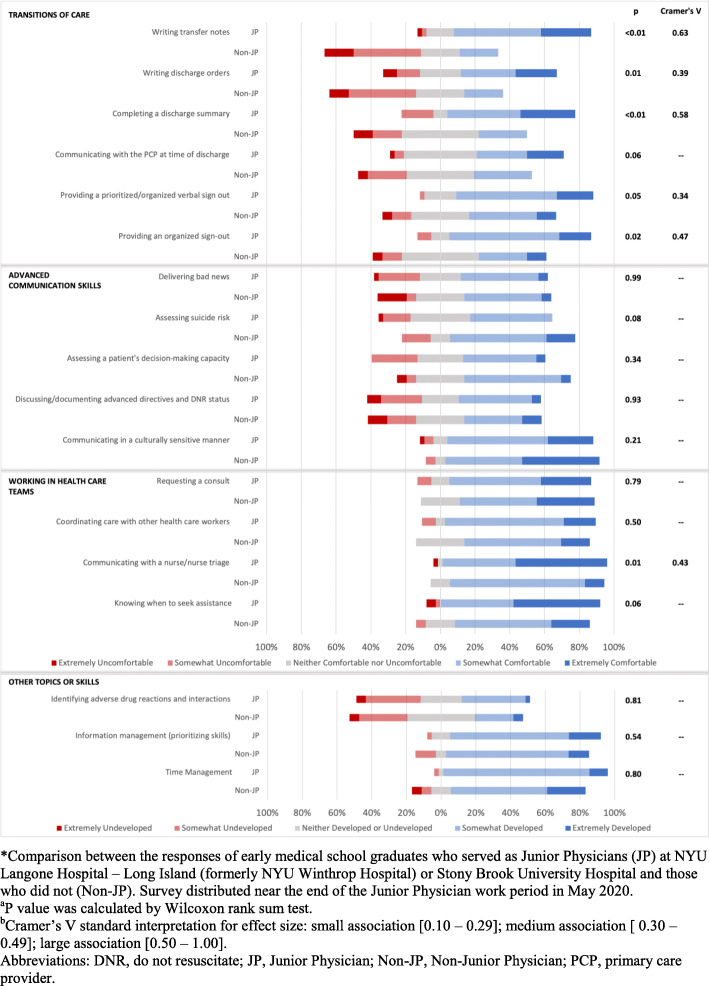
Complete Intern Skills Survey Results: Comparison in Ratings of Comfort in Performing Medical Intern-level Skills and Ratings of Perceived Skills Between COVID-19 JP and Non-JP Early Graduates, May 2020. Comparison between the responses of early medical school graduates who served as Junior Physicians (JP) at NYU Langone Hospital – Long Island (formerly NYU Winthrop Hospital) or Stony Brook University Hospital and those who did not (Non-JP). Survey distributed near the end of the Junior Physician work period in May 2020. ^a^P value was calculated by Wilcoxon rank sum test. ^b^Cramer’s V standard interpretation for effect size: small association [0.10–0.29]; medium association [0.30–0.49]; large association [0.50–1.00]. Abbreviations: DNR, do not resuscitate; JP, Junior Physician; Non-JP, Non-Junior Physician; PCP, primary care provider

Results from an analysis of the Intern Skills survey are presented in Fig. 1. In the Transitions of Care grouping, JPs at the end of their experience reported feeling more comfortable in most measures compared to reports of their Non-JP colleagues. In the Working in Healthcare Teams grouping, JPs reported increased comfort in communicating care with nurse/nurse triage (*P* = 0.01) and knowing when to seek assistance (*P* = 0.06), whereas comfort levels in requesting a consult and coordinating care with other health care workers was not different between the groups (Fig. 1). In the Advanced Communication Skills and Other Topics and Skills groupings, JP reports are largely consistent with those of Non-JPs.

**Fig. 2:**
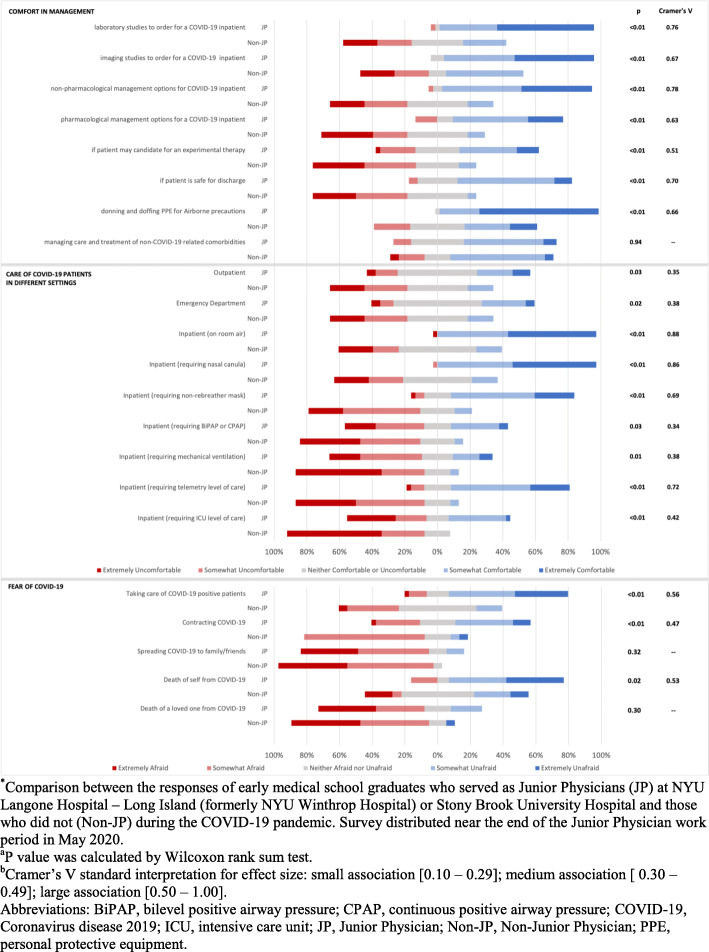
COVID Care Survey Results: Comparison in Ratings of Comfort and Associated Fear in Managing COVID-19 Patients Between JP and Non-JP Early Graduates, May 2020. Comparison between the responses of early medical school graduates who served as Junior Physicians (JP) at NYU Langone Hospital – Long Island (formerly NYU Winthrop Hospital) or Stony Brook University Hospital and those who did not (Non-JP) during the COVID-19 pandemic. Survey distributed near the end of the Junior Physician work period in May 2020. ^a^P value was calculated by Wilcoxon rank sum test. ^b^Cramer’s V standard interpretation for effect size: small association [0.10–0.29]; medium association [0.30–0.49]; large association [0.50–1.00]. Abbreviations: BiPAP, bilevel positive airway pressure; CPAP, continuous positive airway pressure; COVID-19, Coronavirus disease 2019; ICU, intensive care unit; JP, Junior Physician; Non-JP, Non-Junior Physician; PPE, personal protective equipment.

Figure 2 shows data collected by the COVID Care survey. JPs reported increased comfort in monitoring and managing COVID-19 patients than Non-JPs in all settings. Donning and doffing appropriate PPE (97% of JPs vs 21% of Non-JPs, *P* < 0.01) was the area of greatest comfort for JPs. JPs reported lower levels of fear in taking care of COVID-19 patients in addition to contracting and dying from the virus.

Nineteen JPs (49% of eligible JPs) completed the Recap survey and Table 2 reports the results (Supplementary file 4 reports additional Recap survey results). All JPs had direct contact with COVID-19 patients and 15 (83%) reported that a majority of their patients had this diagnosis. The average number of patients that JPs were responsible for caring for increased from three patients in week one to seven patients in week eight. JPs also believed they increasingly contributed positively to the level of patient care and were increasingly helpful to the healthcare team as their work period went on. JPs unanimously reported the belief that the experience will be helpful in residency and beneficial to their training, and all were retrospectively pleased in their decision to work. Eleven JPs (58%) directly interacted with clinical trial personnel in an attempt to enroll a patient in an experimental study for COVID-19 treatment; 10 % of JPs had engaged with clinical trial personnel prior to this experience (*p* < 0.01, Cramer’s V = 0.51). Thirty-seven percent (*n* = 7) of JPs reported a change in their living situation during their work period as a JP due to their position, including moving from the home to minimize infection risk to loved ones (*n* = 6) and moving near the hospital from out-of-town. Zero JPs developed a known COVID-19 infection during the contract period, and none were required to quarantine or stop work due to infection during the work period. Eleven JPs (58%) chose to quarantine away from family for a time period following their work period.

**Table 2:** Recap Survey Responses: COVID-19 Junior Physician Descriptive Characteristics and Opinions of Experience^a^

**Descriptive Characteristics (*n*** **= 19)**			**Value**
**Total Length of Experience, weeks, median [IQR]**			6 [4.0-6.5]
**Hospital Center, n (%)**			
Public (SBUH)			11 (58)
Private (NYUW)			8 (42)
**Direct contact with COVID-19 Patients, n (%), yes**			19 (100)
**ICU days worked, median [IQR]**			
Worked in an ICU (*n*=8)			6 [3.8-12.5]
**Tested positive for COVID-19 since start, n (%)**			
Yes, nasal swab (active infection)			0 (0)
Yes, antibody (prior infection)			3 (17)
**JP Opinions of Experience**			
**Positive Opinion, n (%)**		**Negative Opinion, n (%)**	
Helpful for residency	19 (100)	Harmful for residency	0 (0)
Pleased in choice to work	19 (100)	Regret choice to work	0 (0)
Beneficial to training	19 (100)	Not beneficial to training	0 (0)
Had appropriate supervision	13 (68)	Lacked appropriate supervision	6 (32)
Had appropriate PPE	15 (79)	Lacked appropriate PPE	4 (21)

*Abbreviations*: *COVID-19* Coronavirus disease 2019, *ICU* intensive care unit, *IQR* interquartile range, *JP* junior physician, *NYUW* NYU Langone Hospital – Long Island, *PPE* personal protective equipment, *SBUH* Stony Brook University Hospital

^**a**^Includes a sample of early medical school graduates who worked as Junior Physicians at NYU Langone Hospital – Long Island or Stony Brook University Hospital during the first peak in cases in the COVID-19 pandemic in New York State

Low levels of burnout were reported by both groups. Zero individuals in the Non-JP group reported feelings of burnout whereas two JPs (11%) reported feelings of burnout (P=NS).

### Focus group

Nineteen JPs took part in the Recap survey and were eligible for enrollment in a focus group, in which seven participated.

Table 3 presents the frequency with which each of 16 themes emerged across the focus group along with selected quotes. Themes were placed into four primary categories: development of skills (clinical trials and preparation for residency), patient care (patient, family, and staff communication and delivery of care), safety (JP supervision and PPE availability), and wellness (JP support from colleagues, staff, family and others).

**Table 3:** Grouped themes and selected quotes from a focus group of COVID-19 JPs after the work period, June 2020^a^

Theme	No.^b^	Selected quotes
Development of skills		
Learned the clinical research process in a collaborative setting	16	“The [study team] was nice and professional…we developed a good rapport” “was my first time interacting with study team…extremely easy…[learned] about inclusion and exclusion criteria”
Increased likelihood of engaging patients in trials	5	“I will feel more comfortable pushing for…clinical trials for my patients later on”
Exposed to a new clinical dimension with increasing comfort	25	“Before this,…I had no idea how I would function as an intern. Now, at least, I have a gist of what my role is and how I will fit into the puzzle of my team”
Improved readiness and decreased fear for residency	26	“the experience helped me feel more prepared going into residency…most helpful [were]…learning [the EHR]…to prescribe and knowing that pharmacy will review…learning to communicate with nursing…[it’s] going to be useful.”
Patient Care		
Complex logistics challenged communication with patients and family	16	“we were told not to go into the [patient] room…[and to] call them instead... some patients I never saw…I had trouble calling the family and being a comforting source of info without knowing what the patient looks like”
Patient care improved due to synergy within healthcare team	35	“the nurses…[who] were going in and out of the rooms were helpful...every morning we would talk about how the patients were doing…I got a much better feeling…I now had [multiple] points of reference to [help] determine [my plan]…[and] use to talk to the family about their loved one”
Comfort with provider actions in EHR is paramount to success	11	“I got to put my first order in on an EHR that I was used to and for a patient that I had a lot of supervision on. It was really helpful to build my confidence...[I] feel a little better going into residency”
Caring for very sick patients was challenging^c^	24	“The ICU was the most challenging aspect of it…even on a normal day outside of COVID [it is] outside of the scope of what a lot of us did in…our rotations. But paired with COVID…to start your career taking care of the sickest patients that some doctors with long careers have seen was daunting”
Safety		
Supervision appropriate in most circumstances	28	“I could turn around and ask either one of the interns or the seniors any questions…they were very very willing to help me out and get me acclimated”
Level of responsibility in the hospital was appropriate	12	“I personally felt the level of responsibility…was the same as I will have in a few weeks as an intern…I thought it was appropriate”
Administration receptive to hear and address novel challenges	12	“it was expected that I care for 7 patients on my first day…it changed after that day to having 2 JPs and a resident on each team… [which] was much better.”
Rarely lacked appropriate PPE though difficult to obtain	21	“the only issue that came across was gowns. We were given non-plastic gowns...other than that, we had many face shields, lots of masks” “N95s were not readily available to us but were easily given to us if we asked”
Wellness		
Time for self-reflection in emotionally charged setting is imperative	22	“the…emotional toll of caring for these incredibly sick patients was very difficult and tiring. It was nice to have five days off to…process that all”
Camaraderie with peers and residents was invaluable for personal wellness	20	“One of the greatest things was our attitude and willingness to help each other”
Feelings of appreciation as healthcare providers helped tremendously	18	“I felt more appreciated as a JP than I ever felt as a medical student. For example, the food was provided super often and everyone was very helpful”
Significant life alterations due to contact with contagious patients	10	“we ended up getting married [early]…and, he’s been living with my sister … and I was alone in the apartment and not putting my family at risk”

*Abbreviations*: *COVID-19* Coronavirus disease 2019, *EHR* Electronic health record, *ICU* intensive care unit, *JP* Junior Physician, *PPE* personal protective equipment

^a^Focus group occurred virtually in June 2020 with Junior Physicians who worked at NYU Langone Hospital – Long Island or Stony Brook University Hospital after they had finished their work period

^b^Number of instances where thematic statements occurred

^c^In wellness category in addition to patient care category

Two individuals (29%) reported inadequate supervision early-on as JPs started in the hospital, but these issues were addressed quickly, and additional supervisory support was added. JPs reported that remaining within the hospital system they trained at as medical students made the transition easier because learning a new electronic health care record system would be challenging and poses an additional barrier to a smooth transition into the role of JP. An orientation consisting of review of expectations, practice simulations, and PPE donning and doffing was effective and allowed early graduates to enter the hospital with increased comfort. PPE was available and consistent with CDC guidelines, but some JPs did not always feel it was appropriate.

External to the documented themes, we discussed the decision-making process that went into deciding to work as a JP. JPs described feelings of fear and mixed levels of family encouragement in their decision-making process. Others described significant life events as a result of deciding to work as a JP, such as getting married early and living outside of home to limit risk of contagion to loved ones. Quotes pertaining to these topics and others are provided in Supplementary file 5.

## Discussion

The results of this investigation show that our early graduate medical students were effectively and safely integrated into our medical center responses at the outset of the COVID-19 pandemic in New York. JPs chose to work for wide-ranging, and overlapping, reasons. The JPs developed increased levels of comfort in tasks that are expected of medical interns and deemed their work beneficial in other ways. The decision to work was not without challenges. Appropriate supervision and availability of personal protective equipment was stressed at times, yet insufficiencies were addressed when brought to the attention of supervisors. Further, personal engagements were also altered, as many JPs or family members changed their living situation as a prophylactic safety measure against risk of infection.

### The decision to work as a junior physician

Understandably, the decision to work in a hospital during this frightening time was an incredibly complex one. Students longed to be helpful in this time of need [10, 22, 23] but had to weigh their personal safety and that of family in an uncertain situation. Students who chose not to work reported health concerns being a primary factor, both in risk of spreading infection to family as well as contracting COVID-19 due to inappropriate PPE. These prevailing feelings have been seen from medical trainees at other institutes, as fear of spreading contagion to family is a psychological hardship for health professionals during this pandemic [22, 24]. Twenty five percent of Non-JPs volunteered in other ways, such as in PPE assembly and food shopping for high risk individuals, consistent with ways medical students have reportedly assisted elsewhere [23]. This shows that even if early graduates were unable to engage as a JP due to a multitude of factors, many still had a strong desire to assist in the pandemic response in other, more feasible settings.

The reasons for deciding to work as JPs were centered around a desire to help in addition to educational and social benefits. However, the ease of making such a decision laid on a spectrum. Some JPs faced family discouragement due to family’s fear of their loved one contracting the virus. Prior reports stated final year medical students worried that being away from patient care for an extended period due to the pandemic would lead to “rusty” clinical skills upon beginning internship [22]. These are consistent with the viewpoints expressed by our students, and many believed that the JP experience could reverse this possibility. Other students looked beyond the immediate future, as one student noted that core to their decision to work as a JP was, “being able to live with my choices five or ten years down the line.” And, some reasons were more tangible – a majority of our students reported that the financial incentive contributed to their willingness to work.

### The experiences of early graduates

At our institutions, JPs served in a role most similar to that of medical interns, but with higher supervision and lower volume and responsibility. Our report suggests that this placement resulted in overwhelmingly positive and beneficial experiences. Thus, we muse that future experiences mirroring this role have potential to be positively received by learners. The roles that early graduates played at neighboring institutions varied widely, as each institution had different needs and had no prior framework to guide best practices in utilizing early graduates. Such roles included placing follow-up calls to discharged patients, assisting in research, updating patient families, and facilitating discharge planning [25, 26]. Several institutions graduated medical students early but chose not to permit them to provide direct patient care to COVID-19 patients, which differed from our institutions [27]. Through anecdotal reports, it appears that many of these experiences were well received as well. Though responsibilities and roles varied, all of these early graduates served vital roles in their institutions.

### Benefits for trainees

Interestingly, by the end of their work period, JPs at our institutions reported significantly increased comfort in transitions of care functions and in working in teams compared to their Non-JP colleagues. We postulate that this intriguing difference resulted from the precise level of responsibility offered to JPs over an extended time period compared to what was offered to Non-JPs. Our JPs functioned nearly at the level of an intern for 1 month or more in addition to a required sub-internship experience undertaken during medical school. On the other hand, Non-JPs only had a single four-week sub-internship experience as medical students. Thus, we believe that timing, quantity, and quality of experience played a role. It has been suggested that achieving all the goals of a modern sub-internship in four-weeks may be incredibly ambitious [28]. Our findings provide evidence for this and suggest that an additional experience to that of a sub-internship may be beneficial.

Further, JPs reported significantly greater confidence and comfort in caring for COVID-19 patients. Though expected, this shows that a short experience in a certain specialty at the level of an intern (and greater than a sub intern) can provide immense benefit, as JPs unanimously reported greater comfort heading into residency. Even those going into other seemingly unrelated fields, such as Obstetrics & Gynecology and Pediatrics, reported greater confidence going into residency.

With this knowledge, we further postulate that providing higher level of experiences for final year medical students (or graduating students early) at their own institutions and treating them as interns in responsibility and compensation for a short time period may greatly improve comfort in the transition to residency in other institutions by lessening the learning curve. Such similar experiences occur for final year medical students in New Zealand where students receive a partial training subsidy to serve as “junior interns”; they are expected to act as a physician in all terms except for fully obtaining the degree [29]. Outside of a pandemic, this would be an environment with significantly more supervision than is feasible at the start of internship since the prior-year interns remain at the institution. This could also improve patient safety, as incoming interns may have a better understanding of their knowledge base and role when entering their new institutions.

### Lessons for the future

Through this emergency response, we gleaned insights to improve on how our medical system could handle this situation if it were to present itself again. First and foremost, the option to graduate early and assist during a crisis must be voluntary, not only in the name but also in the essence. Beginning a physician-level role early is a difficult choice to make and one that should feel completely autonomous. Such roles should also be compensated at the level of a resident, both for the work itself and to offset the risk to trainees and family members. Trainees must be provided with a thorough education on patient care in a pandemic, such as the proper use of PPE and necessary lifestyle changes. As previously postulated, this is important for any new trainees, whether they be medical interns (similar in status to our JPs) or medical students engaging with patients during the pandemic [30].

Additionally, recurrent themes from our analysis suggested that proper scheduling and wellness were vital to success. The scheduling model of five-days-on-five-days-off at one center was cited by numerous JPs as ideal for allowing emotional and physical recovery from challenging days in the hospital when compared to a traditional six-day workweek that was used at the other center. Thus, a focus on wellness is paramount; monitoring and responding quickly to concerns greatly improved the experience.

### Limitations and next steps

Our study provides a snapshot of what the experiences were like for early graduates that worked at our two New York medical centers. As early graduate experiences varied widely based on location and institution, those reported here are not all-encompassing. Further, our methods consisted primarily of invalidated tools, as necessitated by the timeline of the investigation. Future study with validated tools could provide conclusions with increased certainty. In the future, we hope that a more expansive evaluation of the experiences of early graduate medical students who worked during the pandemic will be undertaken, including documentation of impacts the experience had on new physicians starting their residency programs. Such a project would provide wider applicability and further inform our conclusions.

## Conclusions

In our experience, final year medical students were able to graduate 2 months early and be quickly redeployed to successfully assist in the COVID-19 pandemic response effort at the height of need in New York. Early graduates chose to work in this role for a spectrum of reasons, ranging from sense of responsibility to financial incentives. As healthcare providers on the frontlines of the pandemic, JPs overwhelmingly reported a positive experience that they believed would be beneficial to their training and helpful as they begin their residencies in a wide range of fields.

## Supplementary Information

**Additional file 1: Supplementary file 1.** Survey Tools**. Supplementary file 2.** Focus Group Script**. Supplementary file 3.** Early Graduate Volunteering Methods. **Supplementary file 4.** Remainder of Recap Survey Results. **Supplementary file 5.** Additional Selected Quotes From COVID-19 JPs in Qualitative Surveys and a Focus Group of 7 JPs After the Work Experience, June 2020

## Data Availability

The datasets used and/or analyzed during the current study are available from the corresponding author on reasonable request.

## Abbreviations

CDC: Centers for Disease Control and Prevention
COVID-19: Coronavirus disease 2019
EHR: Electronic health record
GME: Graduate medical education
ICU: Intensive care unit
JP: Junior physician
NYUW: NYU Langone Hospital – Long Island
PCP: Primary care physician
PPE: Personal protective equipment
SBUH: Stony Brook University Hospital
SO: Significant other
