# Correction to: Experiences of early graduate medical students working in New York hospitals during the COVID-19 pandemic: a mixed methods study

**DOI:** 10.1186/s12909-021-02577-z

**Published:** 2021-03-17

**Authors:** Harrison D. Pravder, Liana Langdon-Embry, Rafael J. Hernandez, Nicholas Berbari, Steven P. Shelov, Wendy L. Kinzler

**Affiliations:** 1 Renaissance School of Medicine, Stony Brook University, HSC T4-147, Stony Brook, NY 11794 USA; 2 NYU Long Island School of Medicine, NYU Langone Hospital – Long Island, 222 Station Plaza, Fifth Floor, Suite 510, Mineola, NY 11501 USA

**Correction to: BMC Med Educ 21, 118 (2021)**

**https://doi.org/10.1186/s12909-021-02543-9**

Following publication of the original article [1], we have been informed that Figs. 1 and 2 were published in low quality and with duplicate figure legends.

**Fig. 1:**
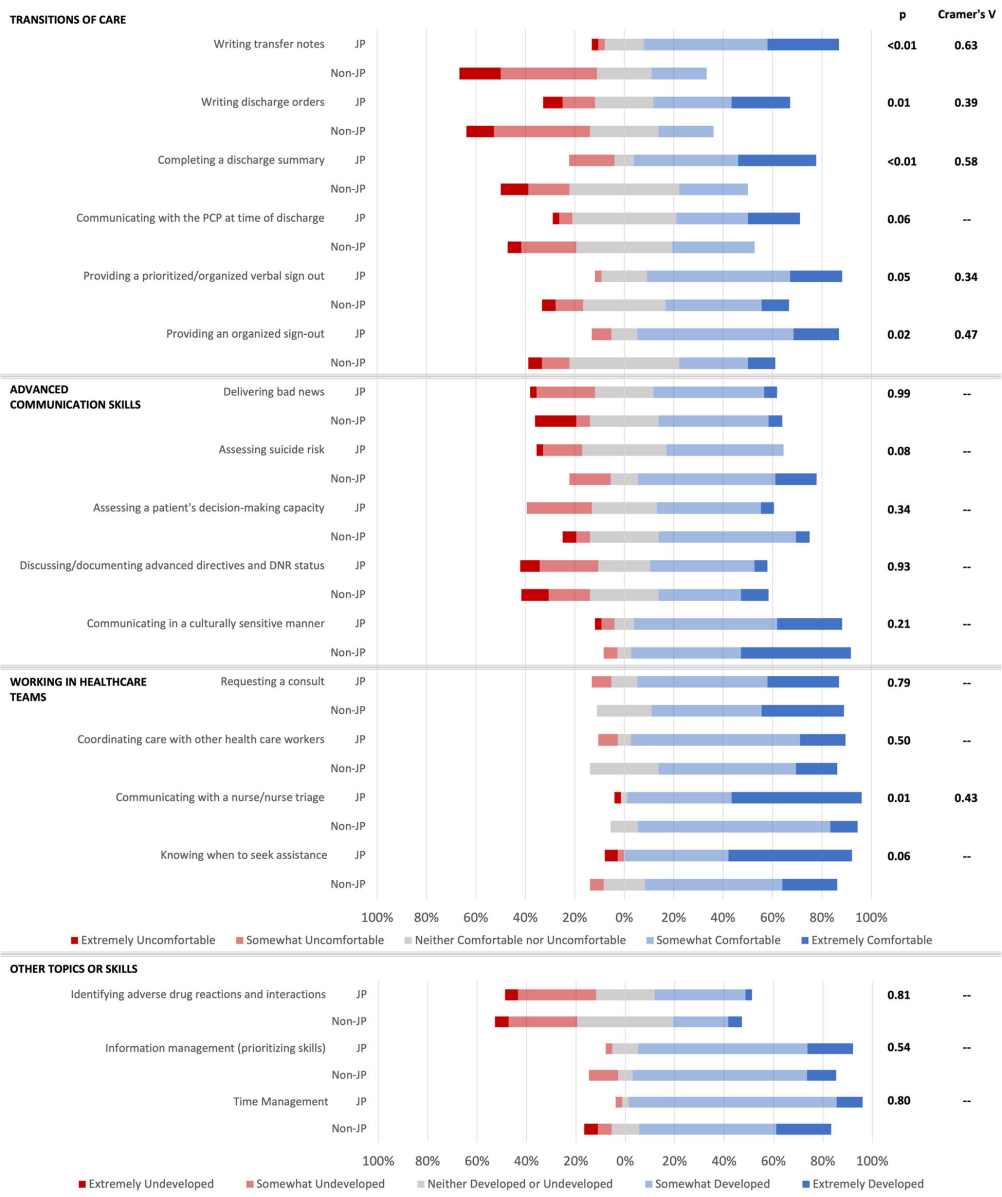
Complete Intern Skills Survey Results: Comparison in Ratings of Comfort in Performing Medical Intern-level Skills and Ratings of Perceived Skills Between COVID-19 JP and Non-JP Early Graduates, May 2020. Comparison between the responses of early medical school graduates who served as Junior Physicians (JP) at NYU Langone Hospital – Long Island (formerly NYU Winthrop Hospital) or Stony Brook University Hospital and those who did not (Non-JP). Survey distributed near the end of the Junior Physician work period in May 2020. aP value was calculated by Wilcoxon rank sum test. bCramer’s V standard interpretation for effect size: small association [0.10–0.29]; medium association [0.30–0.49]; large association [0.50–1.00]. Abbreviations: DNR, do not resuscitate; JP, Junior Physician; Non-JP, Non-Junior Physician; PCP, primary care provider

**Fig. 2:**
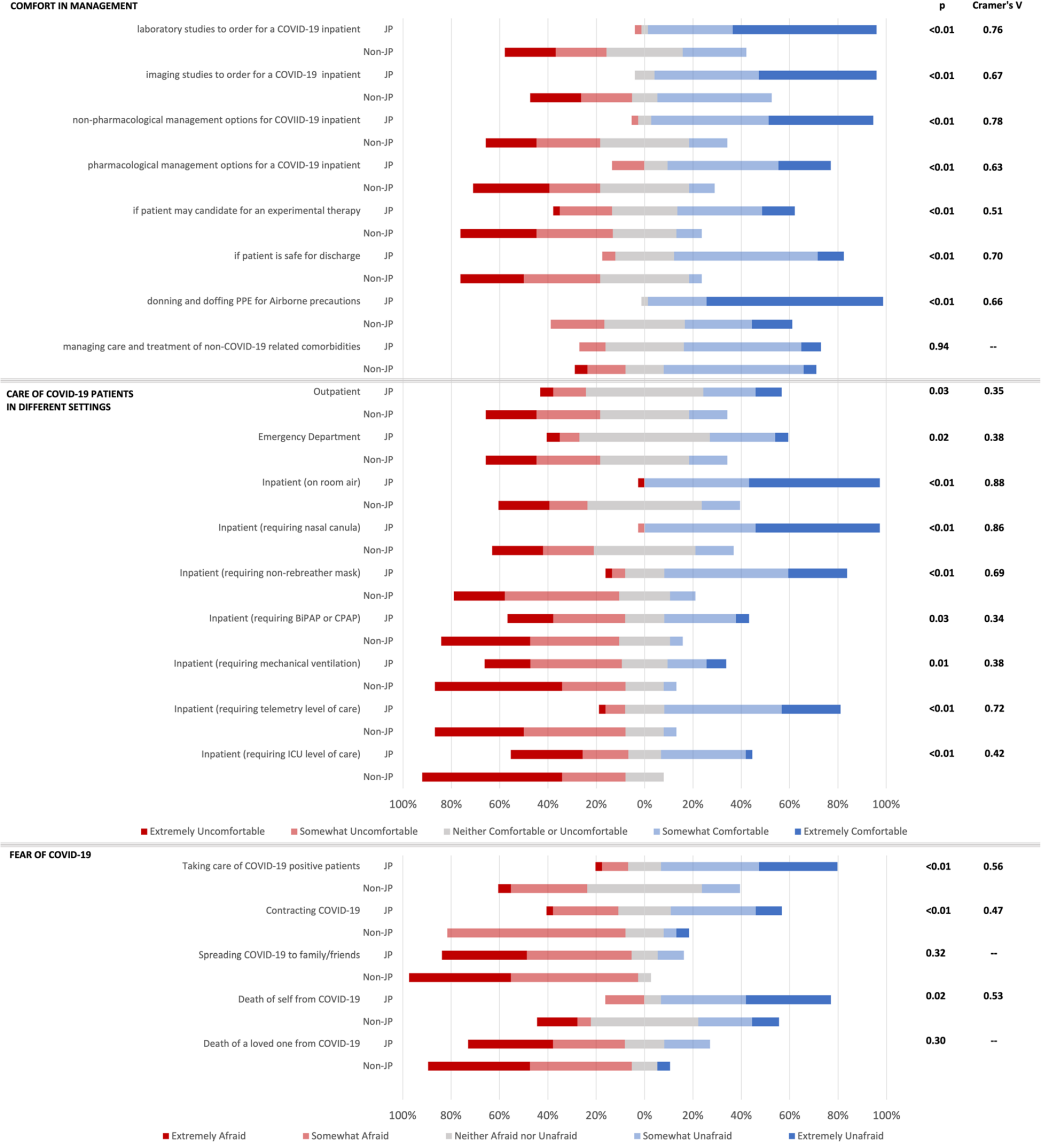
COVID Care Survey Results: Comparison in Ratings of Comfort and Associated Fear in Managing COVID-19 Patients Between JP and Non-JP Early Graduates, May 2020. Comparison between the responses of early medical school graduates who served as Junior Physicians (JP) at NYU Langone Hospital – Long Island (formerly NYU Winthrop Hospital) or Stony Brook University Hospital and those who did not (Non-JP) during the COVID-19 pandemic. Survey distributed near the end of the Junior Physician work period in May 2020. aP value was calculated by Wilcoxon rank sum test. bCramer’s V standard interpretation for effect size: small association [0.10–0.29]; medium association [0.30–0.49]; large association [0.50–1.00]. Abbreviations: BiPAP, bilevel positive airway pressure; CPAP, continuous positive airway pressure; COVID-19, Coronavirus disease 2019; ICU, intensive care unit; JP, Junior Physician; Non-JP, Non-Junior Physician; PPE, personal protective equipment

The original article has been corrected.
