# Supplementary material for: Experiences of early graduate medical students working in New York hospitals during the COVID-19 pandemic: a mixed methods study
Source: BMC Med Educ. 2021 Feb 18;21:118. doi: 10.1186/s12909-021-02543-9 (PMC7891489; doi:10.1186/s12909-021-02543-9)
Supplement: Supplementary file 1 — Additional file 1: Supplementary file 1. Survey Tools. Supplementary file 2. Focus Group Script. Supplementary file 3. Early Graduate Volunteering Methods. Supplementary file 4. Remainder of Recap Survey Results. Supplementary file 5. Additional Selected Quotes From COVID-19 JPs in Qualitative Surveys and a Focus Group of 7 JPs After the Work Experience, June 2020 [file 12909_2021_2543_MOESM1_ESM.docx]

**Supplementary File 1.** Survey Tools

| Question | Answer Options |
| --- | --- |
| Baseline Non-Demographic Survey Aggregate |  |
| Did you work as a physician during the COVID-19 pandemic? (Assistant Physician, Junior Physician, or equivalent – henceforth AP/JP) | Yes or No |
| Did you volunteer in any capacity (outside of being an AP/JP) during the covid-19 pandemic? | Yes or No |
| (If yes) How did you volunteer? | Free Text |
| Why did you decide not to work as an AP/JP? (choose all that apply) | Personal health worries, family exposure worries, lack of PPE worries, work visa issues, time constraint due to residency start date, time constraint due to need of relocating to another state, concern about burnout, concern about lack of appropriate supervision, concern about not being helpful, other (free text) |
| Did you feel that there was pressure for you to work as an AP/JP? | Yes or No |
| Did your family encourage or discourage your working as an AP/JP prior to making a commitment? | Encourage, Discourage, or Neither Encourage nor Discourage |
| (If worked) Did you worry regarding the availability of PPE prior to your working in the hospital? | Yes or No |
| (If worked) Was the provided orientation sufficient in preparing you for your role as an AP/JP? | Yes or No |
| Prior to graduating medical school, have you ever directly contacted a study team in an attempt to enroll a patient you were caring for in an ongoing experimental trial? | Yes or No |
| How comfortable are you in:  Knowing when to seek assistance (“escalating to a senior”)  Communicating with a nurse/nurse triage  Coordinating care with other health care workers  Requesting a consult  Communicating in a culturally sensitive manner  Discussing and documenting advanced directives and do not resuscitate (DNR) status  Assessing a patient’s decision-making capacity  Assessing suicide risk  Delivering bad news  Providing a prioritized/organized verbal sign out  Communicating with primary care physician at time of discharge  Completing a discharge summary  Writing discharge orders  Writing transfer notes | Extremely comfortable, somewhat comfortable, neither comfortable nor uncomfortable, somewhat uncomfortable, or extremely uncomfortable |
| How would you rate your skills in:  Time management?  Information management (prioritizing skills)?  Identifying adverse drug reactions and interactions? | Extremely developed, somewhat developed, neither developed nor undeveloped, somewhat undeveloped, or extremely undeveloped |
| Survey Tools (continued) |  |
| Question | **Answer Options** |
| Baseline Non-Demographic Survey Aggregate (continued)  How comfortable are you in managing a COVID-19 positive patient in the following settings?  Outpatient  Emergency Department  Inpatient (on room air)  Inpatient (requiring nasal canula)  Inpatient (requiring non-rebreather mask)  Inpatient (requiring non-invasive mechanical ventilation, BiPAP/CPAP)  Inpatient (requiring ICU level of care)  Inpatient (requiring telemetry level of care) | Extremely comfortable, somewhat comfortable, neither comfortable nor uncomfortable, somewhat uncomfortable, or extremely uncomfortable |
| How comfortable are you in determining?  The laboratory studies to order for a COVID-19 positive inpatient  The imaging studies to order for a COVID-19 positive inpatient  The non-pharmacological management options for a COVID-19 positive inpatient  The pharmacological management options for a COVID-19 positive inpatient  If a patient may be a candidate for an experimental therapy  If a patient is safe for discharge | Extremely comfortable, somewhat comfortable, neither comfortable nor uncomfortable, somewhat uncomfortable, or extremely uncomfortable |
| How fearful are you of the following circumstances?  Taking care of COVID-19 positive patients  Contracting COVID-19  Spreading COVID-19 to family/friends  Dying from COVID-19  Death of a loved one from COVID-19 | Extremely afraid, somewhat afraid, neither afraid nor unafraid, somewhat unafraid, or extremely unafraid |
| How comfortable are you in…  managing care and treatment of non-covid-19 related comorbidities?  donning and doffing personal protective equipment (PPE) necessary for examine a COVID-19 positive patient? | Extremely comfortable, somewhat comfortable, neither comfortable nor uncomfortable, somewhat uncomfortable, or extremely uncomfortable |
| Using your own definition of burnout, please indicate which of the following statements best describes how you feel about your situation at work? | “I enjoy my work, I have no symptoms of burnout”, “occasionally I am under stress, and I do not always have as much energy as I once did, but I do not feel burned out”, “I am definitely burning out and have 1 or more symptoms of burnout, such as physical and emotional exhaustion”, “the symptoms of burnout that I am experiencing will not go away. I think about frustrations at work a lot”, or “I feel completely burned out and often wonder if I can go on practicing. I am at the point where I may need some changes”. |
| Survey Tools (continued) |  |
| Question | **Answer Options** |
| Recap Survey |  |
| What was the total length of your contract as an AP/JP? | 4, 5, 6, 7, or 8 weeks |
| Do you believe this experience will be helpful for you as you begin your residency? | Yes or No |
| (If yes) How will this experience be helpful? | Free Text |
| (If no) Why do you believe this experience will not be helpful? | Free Text |
| In your opinion, how helpful do you think you were to your medical team at the following times during your AP/JP experience?  Week 1  Week 2  Week 3  Week 4  Week 5  Week 6  Week 7  Week 8 | Extremely helpful, somewhat helpful, neither helpful nor unhelpful, somewhat unhelpful, or extremely unhelpful |
| In your opinion, what impact on the level of care do you believe you contributed to the overall care of your COVID-19 patients at the following time points?  Week 1  Week 2  Week 3  Week 4  Week 5  Week 6  Week 7  Week 8 | Extremely positive, somewhat positive, neither positive nor negative, somewhat negative, or extremely negative |
| Do you believe this experience will be harmful for you as you begin your residency? | Yes or No |
| (If yes) How do you believe this experience may be harmful? | Increased burnout, traumatic experience symptoms, other (free text) |
| Are you glad that you chose to engage in this experience? | Yes or No |
| Was this experience beneficial to your medical training? | Yes or No |
| Did you ever feel that you lacked appropriate supervision? | Yes or No |
| Did you ever feel that you lacked appropriate PPE? If so, please elaborate. | Yes (free text) or No |
| How many days did you work in an ICU in any capacity? | 0 to 50 (analog scale) |
| Did you work with COVID-19 positive patients? | Yes or No |
| (If yes) Were a majority of your patients COVID-19 patients? | Yes or No |
| While working as a JP/AP, did you come into direct contact with COVID-19 positive patients? | Yes or No |
| Have you directly contacted clinical trial personnel in an attempt to enroll one of your patients in a COVID-19 related experimental trial? | Yes or No |
|  |  |
| Survey Tools (continued) |  |
| Question | **Answer Options** |
| Recap Survey (continued)  What was your primary role during the following weeks? (choose the role that you worked most as during the corresponding week, if applicable)  Week 1  Week 2  Week 3  Week 4  Week 5  Week 6  Week 7  Week 8 | DAY – intern level physician (predominantly COVID) or (predominantly non-COVID)  NIGHT – intern level physician (predominantly COVID) or (predominantly non-COVID)  Member of an ancillary team (proning, phlebotomy, PPE, or line)  Other |
| On average, how many hours did you work during the following weeks?  Week 1  Week 2  Week 3  Week 4  Week 5  Week 6  Week 7  Week 8 | 0, 0-19, 19-39, 40-49, 50-59, 60-69, 70-79, or 80+ hours |
| On average, how many patients were you responsible to cover during the following time periods?  Days 1-3  Days 4-7  Week 2  Week 3  Week 4  Week 5  Week 6  Week 7  Week 8 | 0, 1, 2, 3, 4, 5, 6, 7, or 8 patients |
| Approximately how many weeks of **clinical** coursework did you take during the following inclusive time periods?  April 2019-June 2019  July 2019-September 2019  October 2019-December 2019  January 2020-March 2020 | 0, 1, 2, 3, 4, 5, 6, 7, 8, 9, 10, 11, or 12 weeks |
| Have you tested positive for COVID-19 since the start of your AP/JP contract? | Yes, during my contract  Yes, after my contract  No |
| If you have tested positive for COVID-19 since the start of your AP/JP contract, was it an antibody test or nasal swab? | Antibody, Nasal Swab, N/A |
| Have you had to stop working and quarantine yourself while working as an AP/JP? | Yes or No |
| Survey Tools (continued) |  |
| Question | **Answer Options** |
| Recap Survey (continued)  How did your experience differ from what you expected? | Free Text |
| Has your living situation changed as a results of your involvement as an AP/JP? If so, how? | Yes (free text) or No |
| Have you decided to quarantine yourself from family after you completed your AP/JP contract? | Yes or No |
| Did you predominantly work at a public or private hospital? | Public or Private |

**Supplementary File 2.** Focus Group Script

Welcome and thank you for being here today. The purpose of this gathering is to get your feedback on your experiences as Assistant and Junior Physicians during this pandemic. Specifically, we want to understand the aspects of your experience that can be improved should a similar situation present itself in the future.

—Moderator introduces self—

I will guide the conversation by asking questions that each of you can respond to. If you wish, you can also respond to each other’s comments, like you would in an ordinary conversation. It is my job to make sure that everyone here gets to participate and that we stay on track.

This focus group today is anonymous. We will be recording this focus group. The recording will only be used for research purposes and will not be heard by anyone outside of this project.

Firstly, let’s all get acclimated with the Zoom application. In this session we will be using the “thumbs up” reaction often to reflect agreement with a statement. Can everybody find this function in the “reaction” area and select it now.

Great, so let us begin with introductions.

**Q1: Please share with us your focus group name and where you served as a physician. We’ll go alphabetically.**

Now we’ll first proceed to discuss a couple specific topics before opening it up to a wider discussion.

**Q2: Many of you interacted with clinical research teams for the first time during this experience since many COVID-19 specific trials were going on. Firstly, how many of you directly were in contact with a study team regarding a patient in a clinical research study – please raise your hands using the “thumbs up” button.**

Record # ________ out of _______

***Q2a****: Those who interacted with study team members, would you consider your experiences to be positive or negative?*

***Q2b****: Do you believe these experiences will make you more likely to engage patients with clinical research?*

***Q2c****: Anything else to add regarding your experiences communicating with the research team?*

**Q3: Regarding supervision during the experience, it has come to our attention that supervision may have been lacking in certain areas, was this the case for anyone present today?** (Utilize thumbs button)

***Q3a****: (if yes) Can you elaborate on these circumstances?*

**Q3b**: *Did anyone in the administration check-in with you regarding any concerns you may have had?*

**Q3c***: Does anyone have anything else to add regarding supervision?*

**Q4: We have all heard about the troubles in obtaining appropriate PPE for healthcare workers. Let’s go around the room, did any of you care for a covid-19 patient without having appropriate PPE?**

***Q4a****: (if someone says yes) Would you be able to expand on these moments?*

***Q4b****: Does anyone have anything to add on the topic of PPE?*

**Q5: Discuss some of the challenges you faced in this experience. How did you overcome them?**

*--allow up to ten minutes for facilitated discussion—*

*Possible facilitation questions*

1. *How was your communication with patients or their family?*
2. *How was it working with different healthcare workers?*

**Q6: How do you feel this experience has impacted your preparedness for residency?**

*--allow up to TEN minutes for facilitated discussion—*

*Possible facilitation questions*

1. *What specific elements of the experience do you feel will be useful in residency?*
2. *What are some things you learned?*
3. *How has it impacted the level of care you may be able to provide to non-covid patients?*

***Q6a****: Can we get a count? How many believe this experience has made them more prepared to begin residency?*

**Q6b***: Does anyone have anything else to add regarding how this has impacted your preparation for residency?*

**Q7: How could this experience have been made better?**

*--allow up to TEN minutes for facilitated discussion—*

*Possible facilitation questions*

1. *How could orientation have been improved?*
2. *How could you have been more prepared?*
3. *If this were to happen again, how could the hospital do better to prepare graduating medical students?*

**Q8: How was your level of responsibility within the hospital? Was it appropriate?**

*--allow up to FIVE minutes for facilitated discussion—*

*Possible facilitation questions*

1. *Were you treated like an intern?*
2. *Were you treated like a medical student?*
3. *Did you feel useful?*

**Q9: Tell us about your decision to work in this role prior to starting? How did those around you such as family and friends encourage or discourage you prior to making your decision?**

**Q10: Has anything been left unsaid that should have been mentioned? Does anyone have any extra memorable experiences?**

I see our time is up. Thank you so much for sharing this useful information with us. As a token of our appreciation, please accept a $5 e-card.

**Supplementary File 3.** Early Graduate Volunteering Methods

| **Non-Junior Physicians** (n=5)^a^  Food distribution at food bank, PPE assembly, research study  Student-run medical clinic  Helping advise underclassmen how to navigate residency application process  Assembling PPE, grocery deliveries |
| --- |
| **Junior Physicians** (n=6)^b^  Calling patients with COVID-19 test results  COVID-19 data entry in the ED  Donated 3D printer and materials to make mask parts |

^a^one early graduate did not provide a response

^b^three early graduates did not provide a response

**Supplementary File 4.** Remainder of Recap Survey Results


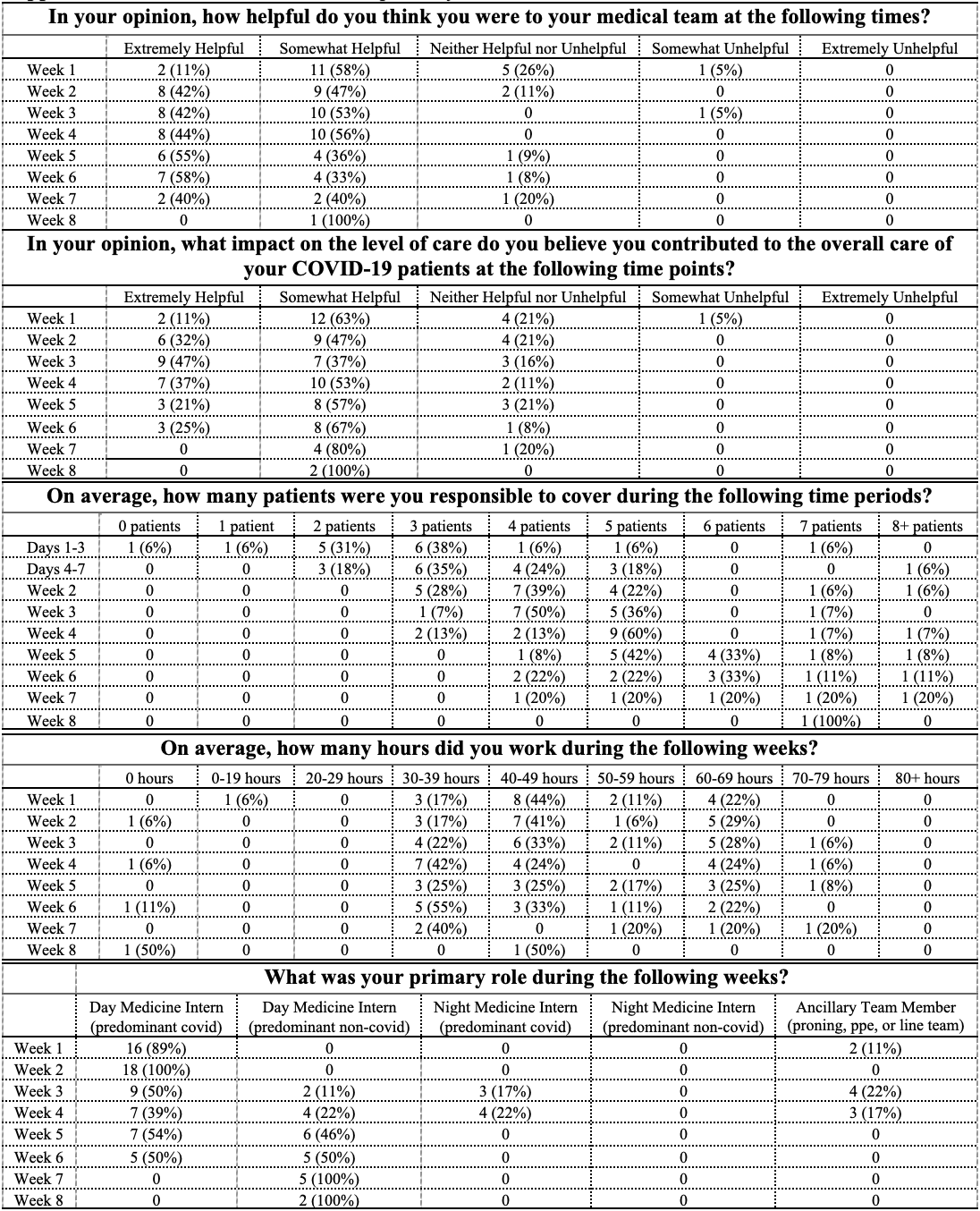


Abbreviations: covid, coronavirus disease 2019 patients; ppe, personal protective equipment

**Supplementary File 5.** Additional Selected Quotes From COVID-19 JPs in Qualitative Surveys and a Focus Group of 7 JPs After the Work Experience, June 2020

**Family Encouragement**

“I had a very strong discouragement from my family, which I understood. There was a lot of fear involved with those conversations and unfortunately it was a unique situation for everyone, so, despite that fact, it didn’t sway me. It was tough to overcome those fears myself.”

“I had probably the opposite experience. I think it wasn't even really a decision for me – the only concern was that I lived in a small apartment my husband, and I didn't want to put him at risk, but he was extremely encouraging as was my entire family. And so, we were able to make it work, and I'm very very glad that we did.”

“I had a similar experience to that. I very strongly wanted to do it and my family knew that it was something that I was going to do and just supported me through that. The concern again, also, I was staying in a small apartment with my then fiancé…he's been living with my sister for the past few months, and I was alone in the apartment and not putting my family at risk. But they were all very supportive and super helpful through the whole thing.”

“I was like in the middle of the two. My family was very much – they took a step back and said, ‘whatever you feel like you want to do, we support you either way.’ Then, for myself, it was a struggle between ‘do I feel ready to do this’ and ‘will I ever actually be ready to do this’. I just ended up making the decision that I knew in the future…I could look back and live with the decision that I've made. But it was a concern because I lived in the basement where my parents live. And, my parents are older, so I had a very long conversation of how I have to quarantine myself and not do certain things and wear mask and wash all these surfaces. But, in the end when I told them what I wanted to do, they were very supportive of the whole thing.”

**Significant Life Changes and Challenges**

“We ended up getting married, so he could have health insurance. And he's been living with my sister for the past few months, and I was alone in the apartment and not putting my family at risk.”

“I started in the ICU, which was just like trying to jump on a train at 60 miles an hour from 0, so that was that was difficult. What I did to overcome, I think I was just patient with myself. I just had to keep realistic expectations on myself, which I think is the most important part. And to take it sort of moment by moment and ask questions that are thoughtful and try not to repeat questions. And take notes and just try to do your best and maintain a positive attitude.”

“The kind of emotional toll of caring for these incredibly sick patients was very difficult and very tiring. It was really nice to have five days off to process that all. And, as a result, now that I have time off before my residency, I don't feel burned out or exhausted from that experience because I had so much break time during it.”

“We had a four on, two off system, which typically worked fine but a lot of times people end up on 6 or 8 days straight. Then, they only had one or two days to recover which felt particularly arduous. And, especially considering what my colleagues said about how difficult emotionally it was to care for these patients and the length of the shifts, if this were to happen again, I think the five on, five off model is much much improved.”

“I spent 2 weeks in the MICU, [and] I worked six days in a row both weeks [with] one day off. So, even if I had time to “utilize the [wellness] resources”, I don't feel like personally I did. Because, I got home really late, [ate] dinner, and then [went] to bed and woke up the next day and did the same thing. I think on my third day, I had one or two of my patients die in succession, so it was a lot to wait until the end of the week to process all of that because you just couldn't do it at work. And then you couldn't do it when you got home because you had to go to sleep and then get up the next day. So, I think that was like only thing I would change. But, a lot of the attendings and residents did reach out and make sure that I was ok with having had one of my patients die, so I think that was helpful. But in terms of like giving time – just maybe going to collect yourselves or being able to speak to somebody would have been nice.”

**Personal Protective Equipment**

”In terms of N95 masks, we were expected to use them for a week – technically for four 12-hour shifts. However, I did have one or two times where I had to go to multiple nurse managers’ offices to get a new mask and was given a hard time. Although, I have to say, the chief residents were great advocates in terms of masks. The only real issue that came across was the gowns. We were given non-plastic gowns, gowns that ripped easily, gowns that pretty easily fell apart in the room, and I heard many nurses and providers joking about how it is just better to go into the room in your scrubs since the gowns were just not effective. Other than that, we had many face shields, we had lots of surgical masks for outside of the rooms, and all of that was sufficient. It was mostly the gowns that were an issue.”

“The N95s were not readily available to us, but they were easily given to us if we asked. I would also say…that the residents were very helpful in getting those. Especially after participating in a code – every time I would be in a code, the resident called me and asked me what size mask I was so they can find another one for me”

“For the most part, we were using plastic gowns…then, we ran out of plastic gowns and for about half a shift, we were told that anyone who needed to go into the rooms needed to wear a garbage bag over their scrubs. Then, they switched that because everyone was very unhappy about that. Then, they just had those yellow flimsy gowns unless you were in an ICU or aerosolizing procedures and stuff like that. But they seemed to stay together ok. I don’t think we had as many falling apart problems”

“face shields and gloves were not ever an issue”

**Physician Supervision**

“I struggled with a lack of supervision…in the 1st deployment. I started my first day working as a JP and it was me and my other resident…it was just two of us and an attending physician for 15 patients, so it was expected that I would take care of seven patients on my first day and that was something that I felt a little unprepared to do…they changed it after that first deployment -- they changed it to having two JPs, a resident on each team, [and an attending]. Then, with this revision it was much better.”

“I have to say I thought that the supervision was outstanding. The way that they had it set up was that each floor typically had 3 teams, two of which were usually intern-based and then one, which was a senior and two JPs …to help to alleviate some of that workload whichever team they joined. I just felt that at any time from my first day to my last day, I could turn around and ask either one of the interns or the seniors any questions that I had and whether it was their team or not, they were very very willing to help me out and get me acclimated. That made it feel very safe which was really wonderful.”

“We were able to do a couple things by ourselves but knowing that we also had the ability to reach out to our intern or a senior for advice…I never found prescribing something without knowing or without being able to just quickly ask about dosages or when to prescribe something or how to look it up. Also, with making phone calls with families, before I made my first phone call - I was…given a rundown of certain key things that I should mention and the way that I should approach the phone call, which made me feel more comfortable, especially considering that it was the first time I was doing that. Also, it helped me for the end of my experience as a JP when I felt comfortable doing it myself.”

“[Supervision] varied wildly week to week depending on who my resident was and who my attendings were. So, I think the trend that I seem to notice was that if the attending was not an internal medicine teaching attending, they tended to be a lot more hands-on and really wanted to be much more involved in knowing what's going on. And, being updated on everything. And I think, a couple of times, they were sitting in the room with us all day where normally it's like the resident in the room and you text the attending on teaching teams. There were a couple times when the non-teaching attendings would just sit in the room with us to…be as involved as possible.”

**How will this experience be helpful for you as you begin your residency?**

“I have learned important skills like how to talk with family members, how to communicate with nurses, how to input basic orders, how to call consults, and how to communicate with other physicians/providers. It also improved my note writing,”

“Understanding flow of hospital as an intern, practice calling consults, basic management of patients and placing orders”

“It allowed me the responsibility of independent patient care.”

“I was able to familiarize myself with the EMR and how to put in orders. I carried multiple critically ill patients and learned how to prioritize. I learned how to work as part of a team and grew comfortable discussing plans of care/next steps with nurses, attendings, residents, and family members.”

“Prepared me for the little orders that interns need to take care of that I didn't realize while in medical school.”

“Useful background for managing medicine patients. General understanding of what it’s like to be a medicine intern”

“Prepare me for COVID-19 patients and residency in general”

“We learn more about taking the textbook learning into real life management of patients for pushing treatment plans forward, managing discharge plans, and how medicine fits into real life situations”

“Time management, workflow, increased confidence, and it was a good overall refresher in basic medical management of common medical problems”

“Increased comfort level with EMR, placing orders, family communication, goals of care conversations, working with nurses in the role of a resident”

“EMR orientation, learning to be main provider for 3-5 patients, familiarizing myself with medicine team flow and interdisciplinary care”

“Got good at putting in orders in the EMR, time management with many patients”

“Learned to use Epic EMR and will be less of a shock getting started in July”

**How did your experience differ from what you expected?**

“I never thought about the fact that on many days, I would be the only “physician” examining a patient since we only had one provider see a patient on a given day to limit contact for infectious reasons. It gave me a real sense of responsibility.”

“Very fulfilling beyond expectations”

“I found stepping into it much easier than expected, the workload was very manageable, and the hospital was extremely respectful of myself and my role.”

“I thought we were going to be “helping out” by taking a couple of patients when needed. But we were functioning as full interns. It wasn’t bad, it just really pushed me to rise to the challenge. I worked hard and so did everyone else but at the end of the day and at the end of my contract I really felt like I helped and made a difference. Also, there were a lot of tough conversations/calls with the family that I had to initiate as the primary intern. I wouldn’t trade the experiences for the world but there was a lot of loss experienced in the ICU setting that was hard to process immediately.”

“It was less stressful than I expected and less high acuity. Well organized and I liked it more than anticipated.”

“less supervision than expected”

“Predominately COVID patients— however, I felt extremely well supported during this hectic time, which was something I did not expect but very much appreciated”

“Patients were much sicker”
